# Autoimmune Diseases Induced or Exacerbated by COVID-19: A Single Center Experience 

**DOI:** 10.1155/2022/9171284

**Published:** 2022-09-06

**Authors:** Yuval Ishay, Ariel Kenig, Limor Rubin, Oded Shamriz, Fadi Kharouf

**Affiliations:** ^1^The Institute of Gastroenterology and Liver Diseases, Department of Medicine, Hadassah Medical Organization, The Faculty of Medicine, Hebrew University of Jerusalem, Jerusalem, Israel; ^2^Department of Medicine, Hadassah Medical Organization, The Faculty of Medicine, Hebrew University of Jerusalem, Jerusalem, Israel; ^3^Allergy and Clinical Immunology Unit, Department of Medicine, Hadassah Medical Organization, The Faculty of Medicine, Hebrew University of Jerusalem, Jerusalem, Israel; ^4^The Lautenberg Center for Immunology and Cancer Research, Institute of Medical Research Israel-Canada, The Faculty of Medicine, Hebrew University of Jerusalem, Jerusalem, Israel; ^5^The Rheumatology Unit, Hadassah Medical Organization, The Faculty of Medicine, Hebrew University of Jerusalem, Jerusalem, Israel

## Abstract

The association between infectious diseases and autoimmunity has long been reported. Specifically, during the coronavirus disease 2019 (COVID-19) pandemic, this relation was further emphasized. The interplay between the two disease processes remains interesting, yet incompletely defined. Herein, we report a case series of six patients presenting with autoimmune phenomena first developed or exacerbated following severe acute respiratory syndrome coronavirus 2 (SARS-CoV-2) infection. We describe the disease course and discuss the possible mechanisms underlying the association between autoimmunity and COVID-19.

## 1. Introduction

Infections may function both as inducers and inhibitors of the development of autoimmune diseases (AIDs). [1] While the exact etiology for this association remains unclear, various theories, such as bystander effect and molecular mimicry, have been suggested for infection-induced autoimmunity [1]. It seems that a multifactorial confluence of hormonal effectors, environmental insults, genetic predisposition, and a putative proximal inciting event are required to induce AIDs. An infectious agent is often suspected to be the latter.

Coronavirus disease 2019 (COVID-19) pandemic has affected millions of lives, including direct impact on morbidity and mortality of infected patients. Corresponding with current studies regarding COVID-19, evidence suggestive of a possible role of severe acute respiratory syndrome coronavirus 2 (SARS-CoV-2) as an inducer of AIDs has accumulated [2, 3]. Indeed, various AIDs were reported to be linked to COVID-19, such as autoimmune hemolytic anemia (AIHA) [4, 5], autoimmune thrombocytopenia [6, 7], and diabetes mellitus [8].

Here, we present our single-center experience with 6 patients with AIDs, developed or exacerbated shortly after or during infection with SARS-CoV-2. Analysis of these patients will add for the better understanding of the association between COVID-19 and autoimmunity.

## 2. Materials and Methods

### 2.1. Study Design

This is a retrospective analysis of computerized medical records of adult patients, who were admitted to the department of medicine or were treated as outpatients of the rheumatology clinic at Hadassah Medical Center, Jerusalem, Israel. All patients were diagnosed and treated during the period of March 2020 and January 2021. Data regarding diagnosis, disease course, treatment, and outcome were retrieved from the patients' files and summarized.

### 2.2. Inclusion and Exclusion Criteria

Adult patients (>18 years), who had fulfilled clinical criteria for a new-onset or exacerbating AID and had a recent or concomitant diagnosis of COVID-19 were included in study. Clinical international diagnostic criteria for Henoch–Schönlein purpura (HSP) [9], polymyalgia rheumatica (PMR) [10], idiopathic thrombocytopenic purpura (ITP) [11], granulomatosis with polyangiitis (GPA) [12], and cryoglobulinemic vasculitis (CV) [13] were used for inclusion of the patients. Supporting laboratory evidence, such as the presence of cytoplasmic anti-neutrophil cytoplasmic antibodies (c-ANCA) in GPA and compatible histopathologic picture, when available, was used to confirm the diagnosis. Cases with insufficient data documentation or where a temporal association between the AIDs and COVID-19 could not be established (for instance due to a prolonged lag between infectivity and the development of autoimmunity) were excluded from the study.

### 2.3. Diagnosis of COVID-19 and Laboratory Workup

COVID-19 was diagnosed by a positive polymerase chain reaction (PCR) from oropharyngeal or nasal swabs, as well as the presence of COVID-19-specific immunoglobulin (Ig) G in the patient sera. Reference ranges for laboratory workup results were used according to institutional internal values.

### 2.4. Ethical Review of the Study

This study was approved by an institutional review board of Hadassah Medical Center (HMO-0089-21).

## 3. Results

### 3.1. Characteristics of the Patients

Patient clinical characteristics are presented in Table 1. Data analysis retrieved 6 patients with a mean age of 54.83 (range: 20–76) years. Five out of the six patients were females. All the patients are residents of the great Jerusalem area and were of Jewish ethnicity.

AIDs associated with COVID-19 included HSP patient (P1), PMR-like disease (P2), ITP (P3 and P6), GPA (P4), and CV (P5). Fifty percent of the patients (P1, P2, and P4) experienced a first presentation of AID following SARS-CoV-2 infection, while AID recurrence was observed in the other half (P3, P5, and P6).

### 3.2. Detailed Description of Clinical Presentation and Course of the Patients

#### 3.2.1. Patient 1: HSP

**Figure 1:**
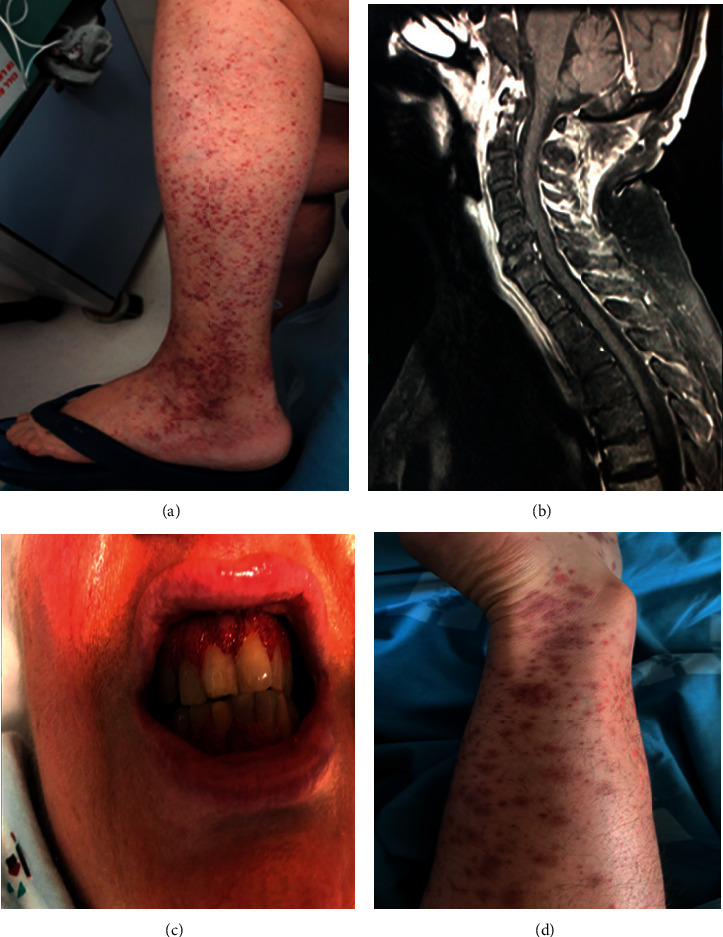
Clinical presentation of patients with autoimmune disease following SARS-CoV-2 infection. (a) Lower limb purpuric lesions in a patient with Henoch–Schönlein purpura. (b) MRI scan in T-1 fat-suppressed sagittal view, showing enhancement and edema in the frontal and posterior cervical paravertebral soft tissues, as well as C2 osteitis, in a patient with a polymyalgia rheumatica-like disease. (c) “Strawberry gingivitis” as a presentation of granulomatosis with polyangiitis. (d) Left lower limb petechial eruption in a patient with cryoglobulinemic vasculitis.

A 20-year-old male was admitted to the internal medicine ward due to skin rash and arthralgia, developing one month following a mild COVID-19. His medical history was notable for Crohn's disease, treated with ustekinumab, with the last dose received 5 weeks pre-admission. The patient‘s current illness manifested with 10 days of migratory arthralgias involving both knees, left wrist, and the left ankle. Besides, a prominent skin rash appeared, with palpable non-blanchable purpura distributed on the lower limbs, buttocks, and arms (Figure 1(a)). Physical examination was otherwise unremarkable. Laboratory evaluation revealed elevated inflammatory markers. Kidney function tests, urinalysis, and chest X-ray (CXR) were all normal. Immune serologies including complement levels, anti-neutrophil cytoplasmatic antibodies (ANCAs), rheumatoid factor, and anti-nuclear antibody were negative. The patient was diagnosed with HSP, developing post-COVID-19. He had a short and uneventful hospital stay, improving spontaneously without the need for systemic treatment.

#### 3.2.2. Patient 2: PMR-Like Disease

A previously healthy 68-year-old female was admitted to the orthopedic surgery department with a 10-day history of occipital headache, shoulder and buttock aches, and prominent morning stiffness. Her complaints appeared one day after recovery from a mild COVID-19. Upon presentation, she was afebrile and had no visual complaints, jaw claudication, temporal tenderness, muscle weakness, or dyspnea. Physical examination revealed a 2/6 systolic murmur, as well as a limited active range of motion in her shoulders and hips bilaterally. Laboratory evaluation was significant for elevated inflammatory markers (Table 1). While computed tomography (CT) scan of the cervical and thoracic spine showed only degenerative changes, a cervical magnetic resonance imaging (MRI) scan revealed edema and enhancement of the paravertebral soft tissues (Figure 1(b)). Blood cultures were sterile, and transthoracic echocardiography ruled out vegetations. A diagnosis of PMR-like disease was made, and the patient was started on prednisone 20 mg, with remarkable recovery. Upon follow-up several weeks following discharge, she was asymptomatic and gradual steroid tapering down was initiated.

#### 3.2.3. Patient 3: ITP

A 36-year-old female, with a previous diagnosis of ITP and known antiphospholipid antibodies, was admitted for gingival bleeding and lower limb skin rash. She reported suffering from an episode of weakness and myalgia, without associated fever or respiratory symptoms, two weeks prior to admission. Her vital signs were normal. Physical examination revealed gingival bleeding and a purpuric rash on her waist and lower limbs. Blood tests demonstrated severe thrombocytopenia, mild leukopenia, and normal CRP. A working diagnosis of ITP exacerbation was made, and the patient was started on intravenous (IV) hydrocortisone treatment. In the search for a potential trigger, a nasopharyngeal swab for SARS-CoV-2 was obtained, returning positive. The patient was transferred to the designated COVID-19 ward, where corticosteroid therapy was continued, leading to a marked improvement in blood counts. Within a few days, she was discharged on high-dose prednisone, with a platelet count of 54 × 10^9^ cells/L.

#### 3.2.4. Patient 4: GPA

A 59-year-old female with a known history of obstetric antiphospholipid syndrome, hypertension, and dyslipidemia presented to the emergency department (ED) complaining of headache, fatigue, and abdominal pain. She had recently been diagnosed with bacterial gingivitis, for which a short course of amoxicillin-clavulanate brought no improvement. One month prior to the patient's presentation, her daughter was diagnosed with COVID-19, to whom she was exposed at that time. Several days post-exposure, the patient suffered from an episode of malaise, ageusia, and anosmia. She was not tested for COVID-19 at that time, and a nasopharyngeal swab PCR was negative for SARS-CoV-2 at the time of her admission. Physical examination showed remarkably swollen and erythematous gums (Figure 1(c)), as well as prominent tenderness over the sinuses and epigastrium. Blood tests revealed mildly elevated liver enzymes, increased amylase, marked leukocytosis, severe anemia, and high CRP and ESR. Additional tests were notable for positive c-ANCA, significantly elevated IgG4 levels, and low haptoglobin (Table 1). A peripheral blood smear was normal. A CT scan of the chest and abdomen demonstrated complicated acute pancreatitis and right subsegmental pulmonary embolism. Considering vasculitis, a gingival biopsy was obtained, displaying fibrinoid necrosis, with no lymphoplasmacytic infiltrate or IgG4 staining. GPA was diagnosed, and the patient was started on a 3-day IV methylprednisolone pulse (500 mg each dose), followed by high-dose prednisone. Full-dose anticoagulation with enoxaparin was also initiated. After initially improving, she sequentially developed right scleritis and right ear mixed hearing loss. The latter necessitated the re-administration of a methylprednisolone pulse. In addition, RTX was used for induction of remission. A few weeks following the index admission, SARS-CoV-2 serology returned positive.

#### 3.2.5. Patient 5: CV

A 70-year-old female, with a previous history of CV, was admitted due to non-palpable, non-blanchable petechial rash, appearing on the lower half of her body (Figure 1(d)). She was receiving maintenance therapy with methotrexate and low-dose prednisone, and her disease was considered quiescent. The rash started several hours prior to her admission and was accompanied by diffuse arthralgia and fatigue. The current symptoms were reminiscent of the presenting symptoms at the time of CV diagnosis. Despite the absence of fever and respiratory symptoms, a screening nasopharyngeal swab returned positive for SARS-CoV-2. Laboratory evaluation showed elevated CRP (Table 1) but was otherwise normal. CXR and urinalysis were unremarkable. The patient was diagnosed with CV flare, and high-dose corticosteroid therapy was started, with prompt improvement.

#### 3.2.6. Patient 6: ITP

A 76-year-old female presented to the ED after falling on her back and right hand. Her medical history was notable for ITP, diabetes mellitus, hypertension, and chronic obstructive pulmonary disease. Due to the incidental presence of cough and mild desaturation, she was tested for COVID-19 by a nasopharyngeal PCR swab and found positive. Apart from the chronic desaturation on room air, her vital signs were normal. While physical examination ruled out gross external bleeding, laboratory results revealed profound thrombocytopenia and anemia. There were no signs of immune-mediated hemolysis; schistocytes were absent on a peripheral blood smear, and direct Coombs test was negative. While initially improving on corticosteroids, platelet counts soon declined once again, necessitating the escalation of the corticosteroid dose and the administration of intravenous immune globulin, yet no improvement was achieved. When a bone marrow biopsy revealed no alternative diagnoses, RTX therapy was initiated, securing a gradual recovery of the platelet counts.

### 3.3. Diagnoses or Exacerbations of Autoimmune Diseases in the Patient Cohort

Clinical symptoms associated with the AIDs are presented in Table 1. Laboratory markers compatible with autoimmunity are presented in Table 1. Elevated acute phase reactants consisted of high C-reactive protein (CRP) in five patients (mean: 15.79 (3.5–24.1) mg/dL; normal range: <0.5 mg/dL). Increased erythrocyte sedimentation rate (ESR) was noted in 2 patients (P2 and P4; 72 and 109 mm/h, respectively; normal range <20 mm/h). Positive c-ANCA and compatible gingival biopsy in P4 both supported the diagnosis of GPA.

### 3.4. Diagnosis of COVID-19

Excluding P4, all patients were diagnosed by a positive PCR test taken from oropharyngeal or nasal swabs during admission. P4 with GPA was diagnosed by COVID-19 positive serum IgG titers.

### 3.5. Treatment and Outcome

All patients, except for P1 (HSP), required immunosuppressive therapy (Table 1), consisting of glucocorticoids, with or without additional immunomodulatory drugs such as rituximab in P4 (GPA). P1 recovered spontaneously. The clinical outcomes were favorable in all of the patients.

## 4. Discussion

In this study, we reported six patients, in which various autoimmune manifestations have appeared de novo or were exacerbated following COVID-19.

Several theories have been suggested to explain the induction of AIDs by different infections. The molecular mimicry theory revolves around the concept that shared structural elements between self-antigens and infectious antigens may drive the immune system to attack the former. Growing evidence supports this theory as human peptide sequences are found to be shared with SARS-CoV-2 proteins, for example, a heptapeptide shared between the human proteome and the viral spike glycoprotein [3]. Some evidence supports this theory, as in the case of acute myocarditis induced by group A *Streptococcus* (GAS) infection due to structural similarities between M-protein of GAS and the myocardium [14]. Indeed, molecular mimicry has been suggested in several cases of COVID-19-related autoimmunity, including endothelial damage and AIHA [15, 16]. Some proposed that the wide distribution of the fusion protein angiotensin-converting enzyme 2 in vascular endothelial cells contributes to SARS-CoV-2 endothelial cell invasion, endothelial damage, and development of vasculitis [17].

Another suggested mechanism is the adjuvant or bystander effect. Proponents of this theory cite the infection-driven inflammatory state as sufficient for providing a “second-hit” for self-antigen recognition by latent immune cells, which are potentially self-reactive. Data suggest that this activation may relate to the innate immune system, allowing for antigen-presenting cells to process self-antigens in the context of “danger signals,” such as toll-like receptor activation [18, 19]. Moreover, SARS-CoV-2 spike protein possesses superantigen activity, enabling broad non-specific T cell activation via MHC class II or the T cell receptor (TCR) and contributing to hyperinflammation and autoimmunity [20].

Lastly, viral persistence, as suggested by its name, is another underlying mechanism. Uncleared infection persisting in affected cells, whether still causing cellular damage or quiescent, may stimulate the immune system, particularly via cytotoxic lymphocytes. Immune cells may continue to mediate viral clearance after the symptomatic infection has resolved, causing inflammation and tissue damage via their cytolytic effects. Considering the known and suspected biology behind COVID-19, this theory may be particularly pertinent. Failed or delayed viral clearance was suspected as the cause for the dreaded immune hyperactivation syndrome associated with COVID-19 and was assumed to play a further role in delayed or prolonged autoimmunity [2]. Moreover, SARS-CoV-2's ability to induce hyperstimulation of the immune response and loss of self-tolerance can lead to the synthesis of multiple autoantibodies with a trigger effect to develop de novo or exacerbate a pre-existing AID [21].

In the context of COVID-19, reports of related immune manifestations have been observed and reported soon after the appearance of the pandemic on the world stage. Hepatitis, myocarditis, and encephalitis, all known to complicate the course of COVID-19, have been postulated to be autoimmune in nature [22–24]. These isolated phenomena may be minor manifestations of the major COVID-19-related syndrome of severe systemic inflammation caused by immune hyperactivation and the hyper-cytokine storm triggered by SARS-CoV-2 infection [15].

Kawasaki-like disease and HSP, which were both long-associated with presumed or proven preceding infections, have been described in post-COVID-19 patients [16, 17]. Guillain–Barré syndrome has likewise been reported [22, 25, 26]. Hematologic conditions, such as ITP and AIHA, and systemic inflammatory diseases, such as systemic lupus erythematosus, have also been described, usually appearing a period after the peak of the viral illness [6, 7].

While strongly suggested, it is too soon to definitively link COVID-19 and autoimmunity in a predictable manner or to define COVID-19 as having a high potential to trigger autoimmunity. However, it will, at the very least, join the host of infectious agents linked to trigger AIDs.

Our study has several limitations, mainly due to its retrospective design and small number of patients. Moreover, a definite cause-and-effect association between SARS-CoV-2 infection and the development of autoimmunity is difficult to establish, since up to date there are no specific laboratory or histological markers. Thus, conclusions from our cohort should be drawn with the proper restrictions. We still believe, however, that our single-center study adds to the growing data regarding COVID-19 in the context of AIDs.

In conclusion, the scope of immune phenomena occurring during and after COVID-19 is wide. Future studies should try to solve the following unanswered issues. Are COVID-19-related manifestations expected to be short-lived? Do they respond to the usual immunosuppressive treatments? Which are the most susceptible populations? Will prompt treatment of COVID-19 reduce the incidence of these illnesses? While answers to these questions remain distant, we believe this pandemic still stands to provide us valuable lessons in epidemiology, immunology, and humility.

## Figures and Tables

**Table 1:** Demographic and clinical characteristics of cases of post-COVID-19 autoimmune diseases

Patient	Age (years)/gender	Time of AID diagnosis following COVID-19 infection (weeks)^*∗*^	Methodology of COVID-19 diagnosis	Autoimmune disease	Episode	Clinical presentation	C-reactive protein (mg/dL)/erythrocyte sedimentation rate (mm/h)^*∗∗*^	Autoantibodies^*∗∗*^	Treatment; outcome
P1	20/M	4	PCR	HSP	First	Migratory arthralgia and purpuric rash	3.5NA	NA	No treatment; resolved spontaneously

P2	68/F	2	PCR	Polymyalgia rheumatica-like disease	First	Headache, shoulder and buttock aches, and morning stiffness	0.20 72	NA	Corticosteroids; clinical improvement

P3	36/F	2	PCR	ITP	Recurrence	Gingival bleeding and purpuric rash	0.33NA	NA	Corticosteroids; rise in platelet count and resolution of clinical findings

P4	59/F	4	Serology	GPA	First	Gingivitis, headache, and abdominal pain	24.1 109	c-ANCA 83.4 U/mL	Corticosteroids and rituximab; impressive clinical recovery and improvement in lab values

P5	70/F	Diagnosed during COVID-19 infection.	PCR	Cryoglobulinemic vasculitis	Recurrence	Petechial rash and arthralgia	7.08NA	NA	Corticosteroids; resolution of symptoms

P6	76/F	Diagnosed during COVID-19 infection.	PCR	ITP	Recurrence	No evident thrombocytopenia-related symptoms	24.1NA	NA	Corticosteroids, IVIG, and rituximab; rise in platelet count

F, female; M, male; P, patient; NA, data are not available; PCR, polymerase chain reaction; HSP, Henöch–Schönlein purpura; ITP, idiopathic thrombocytopenic purpura; GPA, granulomatosis with polyangiitis. ^*∗*^Time since diagnosis was calculated from the first positive nasopharyngeal swab or the suspected initiation of symptoms (the earlier). ^*∗∗*^Laboratory reference normal ranges: CRP: 0–0.5 mg/dL; c-ANCA: <10 U/mL; ESR: 1–20 mm/h.

## Data Availability

The data supporting the results of the study are available from the corresponding author upon reasonable request.

## Abbreviations:

AIDs: Autoimmune diseases
AIHA: Autoimmune hemolytic anemia
ANCA: Anti-neutrophil cytoplasmic antibodies
c-ANCA: Cytoplasmic ANCA
COVID-19: Coronavirus disease 2019
CRP: C-reactive protein
CT: Computed tomography
CV: Cryoglobulinemic vasculitis
CXR: Chest X-ray
GAS: Group A *Streptococcus*
HSP: Henoch–Schönlein purpura
Ig: Immunoglobulin
ED: Emergency department
ITP: Idiopathic thrombocytopenic purpura
IV: Intravenous
MRI: Magnetic resonance imaging
P: Patient
PMR: Polymyalgia rheumatica
RTX: Rituximab
SARS-CoV-2: Severe acute respiratory syndrome coronavirus 2.
